# A Rare Case of Atypical Hemolytic Uremia Syndrome Triggered by Influenza Vaccination

**DOI:** 10.7759/cureus.23577

**Published:** 2022-03-28

**Authors:** Sanjay Kumar, Geeta Bhagia, Jessica Kaae

**Affiliations:** 1 Hospitalist, Benefis Health System, Great Falls, USA; 2 Internal Medicine, Benefis Health System, Great Falls, USA; 3 Internal Medicine, Monmouth Medical Center, Long Branch, USA; 4 Hematology and Oncology, Great Falls Clinic, Great Falls, USA

**Keywords:** hemolytic uremic syndrome, thrombotic microangiopathy (tma), complement, atypical hemolytic uremic syndrome, influenza vaccine

## Abstract

Atypical hemolytic uremic syndrome (aHUS) occurs in patients with defective alternative complement pathways, making them susceptible to thrombotic microangiopathy (thrombocytopenia, intravascular hemolysis, and renal failure), and is usually triggered by infectious agents. Influenza and Streptococcus pneumonia are known triggers for aHUS. However, influenza vaccination triggering aHUS is rarely reported. We present a 30-year-old male who presented with chills, abdominal discomfort, and night sweats after receiving the influenza vaccine. The patient had thrombocytopenia, elevated creatinine, blood urea nitrogen, liver enzymes, and bilirubin with schistocytes with peripheral smear. ADAMTS13 activity was normal so the patient was diagnosed with aHUS. The patient improved with eculizumab and was ultimately found to have a mutation in CD46, which made him susceptible to aHUS. This case shows patients with dysregulated alternative complement pathways may be predisposed to develop aHUS after receiving influenza vaccination.

## Introduction

Hemolytic uremic syndrome (HUS) is a type of thrombotic microangiopathy characterized by thrombocytopenia, intravascular hemolysis, and renal failure [1]. Most commonly, HUS is caused by Shiga toxin produced by Shigella dysenteriae type 1 or Shiga-like toxin-producing Escherichia coli (STEC), called typical HUS or STEC-HUS [2-4]. Secondary HUS is the second type that occurs due to coexisting diseases or conditions [4]. Infections are the most common cause of secondary HUS, which include streptococcal pneumonia and influenza [5-6]. It is also associated with autoimmune diseases [7-8], pregnancy [9], cancer [10-11], solid organ or bone marrow transplantation [12-14], and cytotoxic drugs such as quinine, cyclosporine, tacrolimus, mitomycin C, and many others [15-16]. Atypical HUS (aHUS) is the third type that is associated with genetic or acquired defects in complement activation [4], not associated with existing conditions or infection [17]. However, infections, usually upper respiratory infection, precedes it [18]. In the case of atypical HUS, infection is usually the triggering agent rather than a cause [4].

Many cases of aHUS have been reported, which were triggered by influenza A H1N1 and influenza B infection. We present a rare case of aHUS triggered by influenza vaccination.

## Case presentation

A 30-year-old male with a past medical history of cholecystectomy, anxiety, depression, and daily alcohol use, who three days after receiving intramuscular influenza vaccination, was directly admitted to the hospital from a walk-in clinic due to dysphagia for five days and then developed generalized abdominal discomfort, night sweats, chills, and cola-colored urine for two to three days. Dysphagia started suddenly, mainly with solids. Other symptoms started after he received the vaccination. He mentioned that he also was having intermittent loose stools after cholecystectomy three months ago. The patient denied nausea, vomiting, fever, dizziness, or any other symptoms. Past medical and surgical history include anxiety, depression, biliary dyskinesia, and cholecystectomy in July 2020, respectively. Of note, the patient had previously received an intranasal live attenuated influenza vaccine. The patient was only taking Lexapro 10 mg daily before hospitalization. Family history was positive for grand mal seizure in a brother and leukemia in an uncle. The patient was drinking four to five beers a day and denied smoking and illicit drug use.

On physical examination, his vitals were temperature 99.3 degrees Fahrenheit, blood pressure 143/90 mmHg, heart rate 90 beats per minute, respiratory rate 17 per minute, and oxygen saturation 96% on room air. The patient was awake, alert, oriented, and in acute distress. His pupils were equal bilaterally and reactive, extraocular movements were normal. There was no jugular venous distention. Heart sounds were normal. Lungs were clear to auscultation. The abdomen was soft, non-tender, and non-distended. Bowel sounds were normal. The genitourinary exam was unremarkable. There was no peripheral edema and the neurologic exam was also unremarkable. There were no petechiae or ecchymoses.

The patients' laboratory tests (Labs) on the day of presentation are mentioned in Table 1 and Table 2.

**Table 1:** Blood laboratory results on the day of presentation

Labs	Results	Reference Range	Labs	Results	Reference Range	Labs	Results	Reference Range
White Blood Cell (WBC)	5.8 k/ul	3.6-10.2 k/ul	Chloride (Cl)	105 mmol/l	98-107 mmol/l	Calcium (Ca)	8.2 mg/dl	8.4-10.2 mg/dl
Hemoglobin (Hb)	13.8 gm/dl	14-18 gm/dl	Carbon Dioxide (CO2)	21 mmol/l	22-30 mmol/l	Magnesium (Mg)	2.1 mg/dl	1.6-2.3 mg/dl
Hematocrit (Hct)	36.6%	40%-54%	Aspartate Transaminase (AST)	103 u/l	14-59 u/l	Phosphorus	4.7 mg/dl	2.5-4.5 mg/dl
Platelets (Plt)	26 k/ul	152-348 k/ul	Alanine Transaminase (ALT)	27 u/l	4/49 u/l	Lactate Dehydrogenase (LDH)	5797 u/l	313-618 u/l
Sodium (Na)	135 mmol/l	137-145 mmol/l	Alkaline Phosphatase (ALK)	39 u/l	38-126 u/l	Activated Partial Thromboplastin Time (APTT)	29 seconds	9.3-14 seconds
Potassium (K)	3.6 mmol/l	3.5-5.1 mmol/l	Total Bilirubin	4.9 mg/dl	0.2-1.3 mg/dl	Prothrombin Time (PT)	12.6 seconds	25-36 seconds
Creatinine (Cr)	2 mg/dl	0.7-1.3 mg/dl	Total Protein	6.9 gm/dl	6.3-8.2 gm/dl	International Normalized Ratio (INR)	1.10	
Blood Nitrogen Urea (BUN)	51 mg/dl	9-20 mg/dl	Albumin	3.9 gm/L	3.5-5 gm/dl	Fibrinogen	294 mg/dl	165-432 mg/dl

**Table 2:** Urinalysis and microscopy on the day of presentation *Not Applicable (N/A)

Labs	Results	Reference Range	Labs	Results	Reference Range
Urine Color	Amber	N/A*	Urine Urobilinogen	0.2 mg/dl	<1 mg/dl
Urine Appearance	Cloud	N/A	Urine Leukocyte Esterase	Negative	Negative
Urine PH	6	4.7-7.8	Urine WBC	5/HPF	0-5
Urine Specific Gravity	1.016	1.001-1.030	Urine RBC	5/HPF	0-5
Urine Blood	Moderate	Negative	Urine Squamous Epithelial Cell	Occasional	N/A
Urine Ketones	Negative	Negative	Amorphous Sediment	Present	N/A
Urine Nitrates	Negative	Negative	Urine Bacteria	Negative	N/A
Urine Bilirubin	Negative	Negative	Hyaline Cast	5-10/LPF	N/A
Urine Mucus	Negative	N/A	Urine Myoglobin	1260 MCG/L	Negative
Urine Protein	>500 mg/dl	Negative	Urine Glucose	Negative	Negative

The patient’s abnormal labs tests included Hb 13.1 gm/dl, platelets 26 K/ul, sodium 135 mmol/l, creatinine (Cr) 2 mg/dl, BUN 51 mg/dl, AST 103 U/l, total bilirubin 4.9 mg/dl, indirect bilirubin 2.4 mg/dl, phosphorus 4.7 mg/dl, and LDH 5797 U/l. Urinalysis and microscopy (Table 2) showed moderate blood with only five red blood cells (RBC) per high power field (HPF). Urine myoglobin was 1260 mcg/L. Urine protein was also elevated >500 mg/dl. Urine toxicology was negative. The patients' corrected reticulocyte count was not significantly elevated (1.1%) with an absolute reticulocyte count of 57.6 k/ul. The coronavirus disease 2019 (COVID-19) test came back negative. Peripheral smear demonstrated one to two schistocytes per HPF. Abdominal ultrasound was done due to hyperbilirubinemia and elevated AST, which showed no acute abnormalities. Esophagram done due to dysphagia showed esophageal dysmotility, hiatal hernia, and stenosis at the gastroesophageal junction. Endoscopy was not done initially due to the low platelet count.

**Figure 1:**
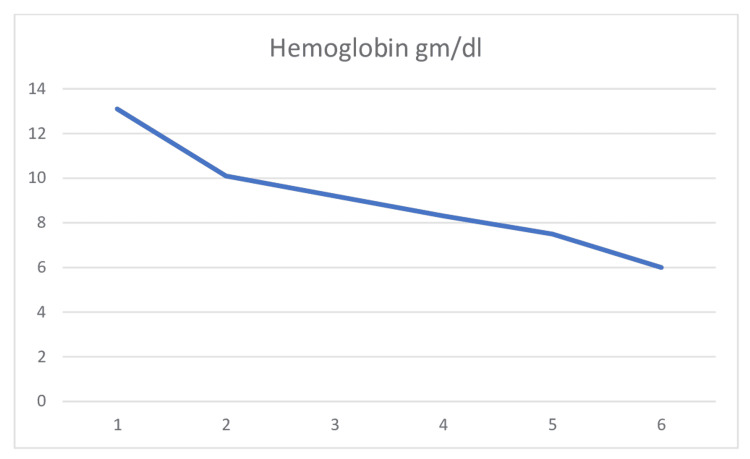
Hemoglobin trend

**Figure 2:**
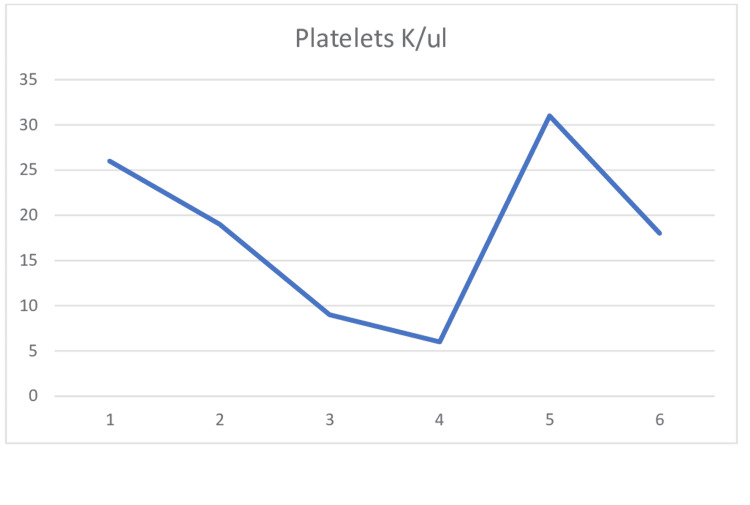
Platelet trend One unit of platelets was transfused when platelets were 6 K/ul, leading to an increase in platelet count.

The patient was started on IV fluids for acute kidney injury. Workup for hemolytic anemia was ordered by a hematologist. His haptoglobin came back low (28 mg/dL). As the coagulation panel and fibrinogen were normal, disseminated intravascular coagulation was ruled out. Vitamin B12 and folate were normal. Autoimmune panels including antinuclear antibody (ANA) and anti-citrullinated antibody (anti-CCP) were negative, as was Coombs' test. HUS was ruled out, as the stool culture was negative. Acute infection of Epstein Barr virus, Cytomegalovirus, human immunodeficiency virus, and hepatitis was negative. To rule out malignancy, computed tomography of the chest, abdomen, and pelvis was done; it was also negative for acute abnormality. Non-tunneled hemodialysis catheter was placed for plasmapheresis. Plasmapheresis and prednisone 1 mg/kg were started for suspected thrombotic thrombocytopenic purpura (TTP) while ADAMTS13 protein was pending. The patients' hemoglobin and platelets continued to downtrend even though the patient was started on plasmapheresis (Figure 1 and Figure 2).

The patient was transfused packed red blood cell (PRBC) when hemoglobin down-trended to 6 g/dl. His ADAMTS13 came back normal. Also, complement CH50, C3, and C4 were normal. Hence, aHUS seemed more likely at this point. The patient was given meningococcal vaccine and started on penicillin VK for prophylaxis along with eculizumab for the treatment of aHUS. The eculizumab dose recommended by the hematologist was 900 mg weekly for four weeks and from Week 5, a maintenance dose of 1200 mg every other week. Plasmapheresis and steroids were discontinued. Platelet count and hemoglobin started improving after eculizumab therapy. Once platelet counts improved, upper endoscopy was done for questionable stricture on esophagogram, which showed mild antral gastritis, no stricture or mass was seen. As Hb, platelets, Cr, AST, and bilirubin continued to improve, he was discharged home with outpatient follow-up with a hematologist.

Genetic analysis was performed as an outpatient; the patient was found to have a heterozygous mutation in membrane complex protein (MCP)/CD46, putting him at high risk of developing aHUS, which was likely triggered by influenza vaccination as other causes were ruled out. Eculizumab was continued. The patient had normal Cr, BUN, AST, bilirubin, platelets, and LDH and improved hemoglobin (Table 3) on the most recent labs.

**Table 3:** Most recent pertinent laboratory results

Labs	Results
White Blood Cell (WBC)	5.9 k/ul
Hemoglobin (Hb)	11.7 gm/dl
Hematocrit (Hct)	34.2%
Platelets (plt)	259 k/ul
Creatinine (Cr)	1.1 mg/dl
Blood nitrogen urea (BUN)	12 mg/dl
Lactate Dehydrogenase (LDH)	446 u/l

## Discussion

Atypical HUS patients have a genetic mutation in complement regulators, leading to impaired regulation of the alternative complement pathway [4]. There is constant mild activation of the alternative complement pathway in the plasma, resulting in the deposition of covalently bound C3b on all the surfaces in contact with plasma [19-20]. If the C3b deposit is inactivated, the surface is not destroyed [4]. If not inactivated, it will lead to the formation of more C3 convertase (C3bBb), which will generate C3b, which may further form C3 convertase leading to its amplification and destruction of cells (even normal cells) by phagocytosis [21]. All the cells in contact with plasma need down regulatory mechanisms to control the alternative complement system, otherwise, it will cause cell and tissue damage, which happens in patients with aHUS [4]. Complement regulators include membrane proteins CD35, CD46, CD55, and CD59 [22]. Factor H, CD35, CD46, and CD55 act as cofactors for factor I in keeping a check on (downregulating) alternative complement pathway by the proteolytic inactivation of C3b, accelerating the decay of C3bBb, competing with Bb in binding to C3b, or a combination of these effects [23]. CD59 acts by stopping the assembly of a membrane attack complex (Figure 1) [4]. Atypical HUS can be caused by many mutations such as impaired recognition of C3b by factor H [24-25], factor I [26], CD46 [27], or disturbed recognition of self-cell surface molecules by factor H [28]. Additionally, autoantibodies against factor H also affect complement regulation in the same way [29]. The C3 convertase half-life is increased, or elimination is prevented with some mutations in C3 or factor B, leading to amplified complement activity [30-31]. Defective metabolism of cobalamin deficiency and Von Willebrand cleaving protease deficiency is also the cause of aHUS [32-34].

**Figure 3:**
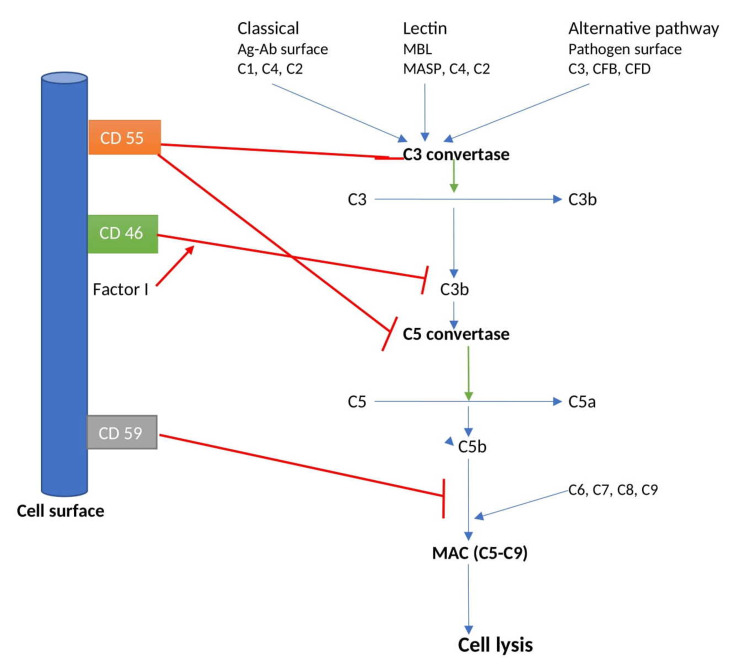
The complement pathway and its regulators

Most of these factors are located on the endothelium. Despite a reduction in one or two factors, complement regulation remains intact [35]. Certain infections in such people can cause inflammation and activation of the dysregulated complement pathway, which may lead to disease process activation [35]. Vascular beds, such as glomerular capillary beds, are at higher risk due to the exposure of the subendothelial matrix to circulatory protein. Their fenestrated endothelium makes those vulnerable to complement attack in patients with defective regulatory factors [35]. The activated complement system damages the endothelium, which will further activate the complement system [4,36]. Also, a prothrombotic state is created due to the exposure of subendothelial collagen, von Willebrand factor, and fibrinogen [35]. Hemolysis occurs due to mechanical damage of erythrocytes from narrowed capillaries from microthrombi and complement-mediated lysis [4].

Influenza A H1N1, seasonal influenza A with or without streptococcal pneumonia co-infection, and influenza B are known to be associated with aHUS [6,33,37-38]. Neuraminidases play a central role in secondary HUS caused by influenza A and streptococcal pneumonia by cleaving sialic acid from glycoproteins on cell surface leading to the unmasking of Thomsen-Friedenreich antigen [39-41]. Influenza A and B may trigger aHUS in a similar manner [37]. Neuraminidase in influenza vaccine can trigger aHUS in susceptible patients, as in ours with MCP/CD46 deficiency.

Eculizumab anti-C5 monoclonal antibody has been approved for treatment since 2011 in America and Europe [42]. Eculizumab decreases complement activation, reduces inflammation, endothelial injury, thrombosis, and renal injury, which will decrease further progression of organ damage in aHUS [43]. ADAMTS13 activity is needed to differentiate between thrombotic thrombocytopenic purpura (TTP) and aHUS, however, the ADAMTS13 activity result takes several days [42]. Therefore, therapy of aHUS with eculizumab is delayed [42]. Patients should receive the meningococcal vaccine at least two weeks prior to receiving eculizumab, as it increases the risk of meningococcal infection [44]. However, most of the time, therapy cannot be delayed for vaccination as patients are acutely sick so two weeks of simultaneous prophylactic antibiotics (penicillin VK 250 mg four times a day, ciprofloxacin 500mg two times a day, or rifampin 600 mg two times a day) are given to the patients [42,44]. In patients who do not show hematologic response to eculizumab therapy after six to eight weeks of therapy, an alternative diagnosis should be considered, for example, diacylglycerol kinase mutation ε (DGKE) presents as an aHUS-like condition without complement activation [44].

## Conclusions

Influenza A and B are known triggers of aHUS in patients with complement deficiency, but the influenza vaccine is rare. Further studies may be needed to stratify such patients to weigh the risks and benefits of influenza vaccination.
